# Supplementary material for: Endogenous activity modulates stimulus and circuit-specific neural tuning and predicts perceptual behavior
Source: Nat Commun. 2020 Aug 11;11:4014. doi: 10.1038/s41467-020-17729-w (PMC7419548; doi:10.1038/s41467-020-17729-w)
Supplement: Supplementary file 1 — Supplementary Information [file 41467_2020_17729_MOESM1_ESM.pdf]

Supplementary Information

Endogenous activity modulates stimulus and circuit-specific neural tuning and predicts perceptual behavior

Li *et al.*

Supplementary Notes

Supplementary Note 1. Classification using only pre-stimulus features

To validate that there was no discriminant information in the pre-stimulus activity, for each of the category-selective electrode, we trained a classifier using only the pre-stimulus activity. Across the 246 electrodes no significant discriminant information was presented in the pre-stimulus activity (average d' = 0.023; $t(245) = 1.44$; $p > 0.1$, paired two-sided).

Supplementary Note 2. Concerns about category-level repetition

A possible confounding factor is the long-lasting activity from the prior trial, likely induced by the one-back task, which has been demonstrated in previous studies[1]. This could become problematic when two consecutive trials shared the same category conditions but did not exactly repeat at the exemplar level. However, as shown in Supplementary Table 1, with category-level repetitions completely removed from the trials, similar modulation effects were still found when comparing the classification accuracy with and without conditioning on the pre-stimulus activity.

Supplementary Note 3. Predicting distance to post-stimulus decision boundary

In addition to the two-stage GLM presented in the main text, a linear regression model was directly applied to evaluate the relationship between pre-stimulus activity and the absolute distance to the decision boundary in the post-stimulus discriminant model. Specifically, we solved the following linear regression problem:

$$\left|X_{evk}\beta_{evk}\right| = X_{pre}\beta_{pre}$$

Similar to the main results presented in Figure 2 and Table 1, we found significant correlation between pre-stimulus activity and absolute distance to the decision boundary in all categories

(Supplementary Table 3). This suggests that the pre-stimulus activity predicts the distance to classification boundary on a trial-by-trial basis.

Supplementary Tables

Supplementary Table 1. The comparisons of classification results from the two-stage GLM when excluding all repeated trials with the same category as the 1-back trial

Category	Bodies	Faces	Words	Tools	Houses	Scrambled non-objects
# of electrodes	9	56	92	16	37	36
d' (evoked only)	1.1018	1.5301	1.0847	0.7881	1.0594	0.8677
d' (evoked + endogenous)	1.1936	1.6091	1.1904	0.8990	1.1948	1.0651
t-stat (paired *t*-test)	1.9225	4.7293	7.3961	4.0891	5.7743	5.8979
p-value (two-sided)	0.0908	1.6×10^{-5}	$< 10^{-5}$	9.7×10^{-4}	$< 10^{-5}$	$< 10^{-5}$

Supplementary Table 2. The comparisons of classification results from the two-stage GLM when only considering electrodes in VTC

Category	Bodies	Faces	Words	Tools	Houses	Scrambled non-objects
# of electrodes	8	56	77	14	37	34
d' (evoked only)	1.1051	1.3957	0.9594	0.6994	1.0585	0.8477
d' (evoked + endogenous)	1.2391	1.5072	1.0951	0.8016	1.2046	1.0442
t-stat (paired *t*-test)	2.5523	4.9466	6.9895	3.1427	5.1421	5.2411
p-value (two-sided)	0.038	$< 10^{-5}$	$< 10^{-5}$	0.0078	$< 10^{-5}$	$< 10^{-5}$

Supplementary Table 3. The R^2 of the linear regression model between pre-stimulus activity and the absolute distance to the decision boundary in the post-stimulus discriminant model.

Category	Bodies	Faces	Words	Tools	Houses	Scrambled non-objects
# of electrodes	9	56	92	16	37	36
R^2	0.0717	0.0507	0.0377	0.0275	0.0361	0.0221
p-value (two-sided)	0.0327	$< 10^{-5}$	2.78×10^{-4}	0.0150	0.0017	0.0678

Supplementary Figures

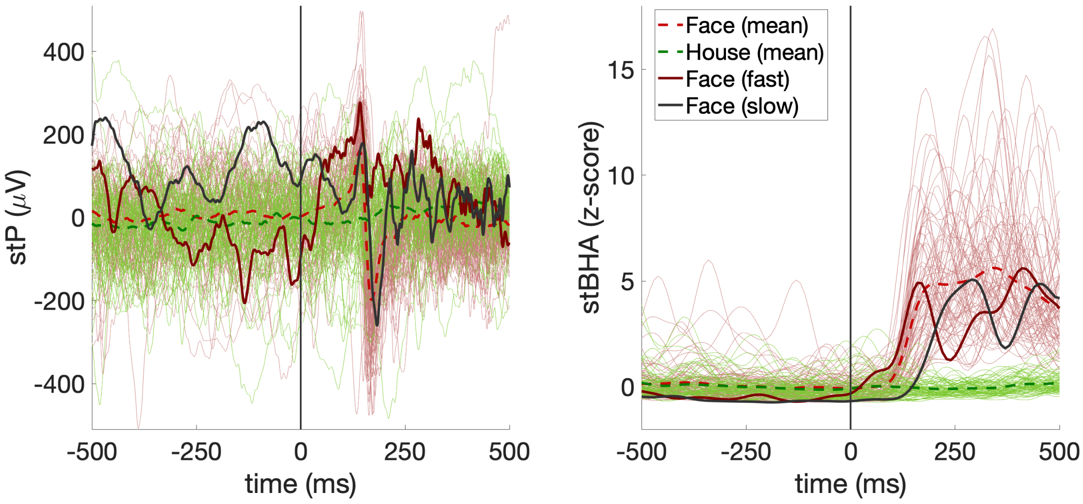

Supplementary Figure 1. Timecourse of stP and stBHA from an example electrode. Single trial field potential (left panel) and broadband high-gamma (right panel) activity recorded from an example category-selective electrode with regard to its preferred condition (faces) and a non-preferred condition (houses). Vertical black line indicates stimulus onset time, single trials are plotted in thin lines, averaged stP and stBHA responses are plotted in dashed lines, dark red line represents a fast response trial (RT = 688 ms), dark gray line represents a slow response trial (RT = 985 ms).

Supplementary Methods

Solving the two-stage GLM using coordinate descent

Solve the elastic-net problem in the first step

In the first step, we set $\beta_{pre} = 0$, and solve the following elastic-net problem, we follow the classical coordinate descent method[2]:

$$\underset{\beta_{evk}}{\text{argmin}} \quad -\ell(\beta_{evk}) + \lambda_1 P_\alpha^{evk}(\beta_{evk}) \tag{1}$$

where β_{evk} is a vector that contains the intercept β_0^{evk} and the feature weights β^{evk}

$$\begin{aligned} -\ell(\beta_{evk}) &= -\frac{1}{N}\{y^T(X_{evk}\beta_{evk}) - \mathbf{1}^T \log(1+\exp(X_{evk}\beta_{evk}))\} \\ &= -\frac{1}{N}\sum_{i=1}^{N}\{y_i(\beta_0^{evk} + x_i^T\beta^{evk}) - \log[1+\exp(\beta_0^{evk} + x_i^T\beta^{evk})]\} \end{aligned} \tag{2}$$

and

$$\begin{aligned} P_\alpha^{evk}(\beta_{evk}) &= \frac{1-\alpha}{2}\|\beta_{evk}\|_2^2 + \alpha\|\beta_{evk}\|_1 \\ &= \sum_{i=1}^{P}[\frac{1}{2}(1-\alpha)\|\beta_j^{evk}\|_2^2 + \alpha\|\beta_j^{evk}\|_1] \end{aligned} \tag{3}$$

For simplicity of the notation, we are omitting the superscript 'evk' in the following part, and we assume that X has been standardized such that each dimension $x_{:j}$ has 0 mean and unit variance.

During the optimizing iterations, assume that the current solution is $[\tilde{\beta}, \tilde{\beta}_0]$, we are solving the updated solution $[\beta_0, \beta]$ following problem:

$$\underset{\beta,\beta_0}{\text{argmin}} \quad -\ell_{\tilde{\beta},\tilde{\beta}_0}(\beta, \beta_0) + \lambda_1 P_\alpha(\beta) \tag{4}$$

we can use quadratic approximation around $[\tilde{\beta}, \tilde{\beta}_0]$ for the negative log likelihood term in (4)

$$-\ell_{\tilde{\beta},\tilde{\beta}_0}(\beta, \beta_0) = -\ell(\tilde{\beta}_0, \tilde{\beta}) - \nabla\ell(\tilde{\beta}_0, \tilde{\beta})^T\Delta\beta - \frac{1}{2}\Delta\beta^T H(\tilde{\beta}_0, \tilde{\beta})\Delta\beta + R(\|\Delta\beta\|^2) \tag{5}$$

$$\approx -\frac{1}{2}\sum_{i=1}^{N} w_i(z_i - \beta_0 - x_i^T\beta)^2 + C(\tilde{\beta}_0, \tilde{\beta}) \tag{6}$$

Note that the last term does not depend on $[\beta_0, \beta]$, and we have gradient and Hessian at $[\tilde{\beta}, \tilde{\beta}_0]$ as

$$\nabla \ell(\beta) = \frac{1}{N} \sum_{i=1}^{N} [y_i - P_{\tilde{\beta}_0, \tilde{\beta}}(x_i)] x_i \tag{7}$$

$$H(\tilde{\beta}_0, \tilde{\beta}) = -\frac{1}{N} \sum_{i=1}^{N} P_{\tilde{\beta}_0, \tilde{\beta}}(x_i)(1 - P_{\tilde{\beta}_0, \tilde{\beta}}(x_i)) x_i x_i^T \tag{8}$$

where $\Delta\beta = [\beta_0 - \tilde{\beta}_0, \beta^T - \tilde{\beta}^T]^T$ is the update difference, and $P_{\tilde{\beta}_0, \tilde{\beta}}(x_i) = \frac{1}{1+\exp(-\tilde{\beta}_0 - x_i^T \tilde{\beta})}$ is the estimated likelihood at $[\tilde{\beta}, \tilde{\beta}_0]$.

Plugging (7),(8) into (5) and the comparing with (6), we get

$$z_i = \tilde{\beta}_0 + x_i^T \tilde{\beta} + \frac{y_i - P_{\tilde{\beta}_0, \tilde{\beta}}(x_i)}{P_{\tilde{\beta}_0, \tilde{\beta}}(x_i)(1 - P_{\tilde{\beta}_0, \tilde{\beta}}(x_i))} \tag{9}$$

$$w_i = \frac{1}{N} P_{\tilde{\beta}_0, \tilde{\beta}}(x_i)(1 - P_{\tilde{\beta}_0, \tilde{\beta}}(x_i)) \tag{10}$$

As a result, solving (4) becomes solving the following regularized weighted least-squares problem:

$$\underset{\beta, \beta_0}{\text{argmin}} \quad -\frac{1}{2} \sum_{i=1}^{N} w_i (z_i - \beta_0 - x_i^T \beta)^2 + \lambda_1 \sum_{i=1}^{T_2} [\frac{1}{2}(1-\alpha) \|\beta_j\|_2^2 + \alpha \|\beta_j\|_1] \tag{11}$$

We use coordinate descent to solve (11). Taking subgradient and set it to 0, through some calculus we get coordinate-wise update

$$\tilde{\beta}_j \leftarrow \frac{S(\sum_{i=1}^{N} w_i x_{ij} (z_i - \tilde{z}_i^{(-j)}), \lambda\alpha)}{\sum_{i=1}^{N} w_i x_{ij}^2 + \lambda(1-\alpha)} \tag{12}$$

where $\tilde{z}_i^{(-j)} = \tilde{\beta}_0 + \sum_{k \neq j} x_{ik} \tilde{\beta}_k$ is the fitted value excluding the contribution from x_{ij}, and $S(z, \gamma) = \text{sign}(z)(|z| - \gamma)_+$ is the soft-thresholding operator, where

$$S(z, \gamma) = \text{sign}(z)(|z| - \gamma)_+ = \begin{cases} z - \gamma & \text{if } z > 0 \text{ and } \gamma < |z| \\ z + \gamma & \text{if } z < 0 \text{ and } \gamma < |z| \\ 0 & \text{if } \gamma \geq |z| \end{cases} \tag{13}$$

To sum up, in the first step, we solve the elastic-net regularized GLM using coordinate de-

scent, as shown in Algorithm 1.

Algorithm 1: Solve the elastic-net regularized GLM using coordinate descent

Data: data matrix $X_{evk} \in \mathbb{R}^{N \times T_2}$ for post-stimulus part of the data, data label $y \in \mathbb{R}^N$; where N is the number of samples, $T_2 = t^P_{evk} + t^{BHA}_{evk}$, and $X_{evk} = [X^P_{evk}, X^{BHA}_{evk}]$;
Parameters: the elastic-net hyper-parameter α, maximum regularization parameter λ_{max} and minimum regularization parameter $\epsilon\lambda_{max}$.
Result: Weight vectors for post-stimulus features $\beta^*_{evk} = [\beta^{evk}_0, \beta^P_{evk}, \beta^{BHA}_{evk}]$

Fit the elastic-net problem for post-stimulus features:
for *the i-th cross-validation split* $\{X^{(i)}_{evk,train}, X^{(i)}_{evk,test}\}$ **do**
    for $\lambda \leftarrow \lambda_{max}$ **to** $\epsilon\lambda_{max}$ *(decrement λ)* **do**
        while *not converge* **do**
            update the current quadratic approximation (11)by computing (9),(10);
            for $j \leftarrow 1$ **to** T_2 *(cyclic coordinate descent)* **do**
                update the weight of each coordinate $\tilde{\beta}_j$ using (12);
        estimate the deviance of the solution for current λ on $X^{(i)}_{evk,test}$;
find optimal λ^* and the corresponding β^*_{evk} that minimizes deviance.

Solve the group elastic-net GLM problem in the second step

The second step of fitting the two-stage GLM requires fixing the contribution from post-stimulus features and optimize the model with group elastic-net penalty on the pre-stimulus features[3]. By fixing the weights from post-stimulus features, for each sample x_i, we get a fixed offset $b_i = \beta^{evk}_0 + x^T_i \beta^*_{evk}$. Therefore, we have

$$\beta^*_{pre} = \underset{\beta_{pre}}{\text{argmin}} \quad -\ell(\beta^*_{evk}, \beta_{pre}) + \lambda_2 P^{pre}_\alpha(\beta_{pre}) \tag{14}$$

where

$$\begin{aligned} -\ell(\beta^*_{evk}, \beta_{pre}) &= -\frac{1}{N}\{y^T(b - X_{pre}\beta_{pre}) - \mathbf{1}^T \log(1 + \exp(b - X_{pre}\beta_{pre}))\} \\ &= -\frac{1}{N}\sum_{i=1}^{N}\{y_i(b_i - \beta^{pre}_0 - (x^{pre}_i)^T\beta^{pre}) - \log(1 + \exp(b_i - \beta^{pre}_0 - (x^{pre}_i)^T\beta^{pre}))\} \end{aligned} \tag{15}$$

and

$$\begin{aligned} P^{pre}_\alpha(\beta_{pre}) &= \frac{1-\alpha}{2}\|\beta_{pre}\|^2_2 + \alpha\|\beta^P_{pre}\|_1 + \alpha\|\beta^{BHA}_{pre}\|_1 + \alpha\mathcal{G}(\beta^{phase}_{pre}) \\ &= \frac{1-\alpha}{2}\|\beta_{pre}\|^2_2 + \alpha\sum_{g=1}^{G}\sqrt{p_g}\|\beta^{(g)}_{pre}\|_2 \end{aligned} \tag{16}$$

where the second term is the group-lasso penalty on the pre-stimulus features. Similarly to the previous part, from now on we omit the 'pre' in superscript and subscript for simplicity.

Similar to previous part, we first take the quadratic approximation of the negative log likelihood at the current iteration step around $[\tilde{\beta}_0, \tilde{\beta}]$ as

$$
\begin{aligned}
-\ell_{\tilde{\beta},\tilde{\beta}_0}(\beta,\beta_0) &= -\ell(\tilde{\beta}_0,\tilde{\beta}) - \nabla\ell(\tilde{\beta}_0,\tilde{\beta})^T\Delta\beta - \frac{1}{2}\Delta\beta^T H(\tilde{\beta}_0,\tilde{\beta})\Delta\beta + R(\|\Delta\beta\|^2) \quad (17)\\
&\approx -\frac{1}{2}\sum_{i=1}^{N} w_i(z_i - \beta_0 - x_i^T\beta)^2 + C(\tilde{\beta}_0,\tilde{\beta})\\
&= -\frac{1}{2}(z - \sum_{g=1}^{G} X^{(g)}\beta^{(g)})^T W(z - \sum_{g=1}^{G} X^{(g)}\beta^{(g)}) + C(\tilde{\beta}_0,\tilde{\beta}) \quad (18)
\end{aligned}
$$

where $z = [z_1, ..., z_N]^T$, $W = diag\{w_1, ..., w_N\}$, and $X = [X^{(1)}, ..., X^{(G)}]$ is the blocks in X that corresponding to each group $\beta^{(g)}$ and

$$
z_i = b_i + \tilde{\beta}_0 + x_i^T\tilde{\beta} + \frac{y_i - P_{\tilde{\beta}_0,\tilde{\beta}}(x_i)}{P_{\tilde{\beta}_0,\tilde{\beta}}(x_i)(1 - P_{\tilde{\beta}_0,\tilde{\beta}}(x_i))} \quad (19)
$$

$$
w_i = \frac{1}{N}P_{\tilde{\beta}_0,\tilde{\beta}}(x_i)(1 - P_{\tilde{\beta}_0,\tilde{\beta}}(x_i)) \quad (20)
$$

and

$$
P_{\tilde{\beta}_0,\tilde{\beta}}(x_i) = \frac{1}{1 + \exp(-b_i - \tilde{\beta}_0 - x_i^T\tilde{\beta})} \quad (21)
$$

Analogously, solving (14) becomes iteratively solving the following regularized weighted least-squares problem:

$$
\operatorname*{argmin}_{\beta,\beta_0} \quad -\frac{1}{2}(z - \sum_{g=1}^{G} X^{(g)}\beta^{(g)})^T W(z - \sum_{g=1}^{G} X^{(g)}\beta^{(g)}) + \lambda_2\frac{1-\alpha}{2}\|\beta\|_2^2 + \lambda_2\alpha\sum_{g=1}^{G}\sqrt{p_g}\|\beta^{(g)}\|_2 \quad (22)
$$

Let $r^{(-g)} = z - \sum_{j\neq g} X^{(j)}\beta^{(j)}$ be the residual excluding the contribution of $\beta^{(g)}$. The first-order optimality condition gives

$$
(X^{(g)})^T W r^{(-g)} + \big[\lambda_2(1-\alpha)I^{(g)} - (X^{(g)})^T W X^{(g)}\big]\beta^{(g)} + \lambda_2\alpha\sqrt{p_g}\nu^{(g)} = 0 \quad (23)
$$

where subgradient

$$
\nu^{(g)} \in \begin{cases} \{\frac{\beta^{(g)}}{\|\beta^{(g)}\|_2}\} & \text{if } \beta^{(g)} \neq 0 \\ \{u \mid \|u\| \leq 1\} & \text{if } \beta^{(g)} = 0 \end{cases} \quad (24)
$$

The optimal solution for each group is given as

$$
\tilde{\beta}^{(g)} = \begin{cases} \Big((X^{(g)})^T W X^{(g)} + \lambda_2\big[(1-\alpha) + \frac{\alpha\sqrt{p_g}}{\|\tilde{\beta}^{(g)}\|}\big]I^{(g)}\Big)^{-1}(X^{(g)})^T W r^{(-g)} & \text{if } \|(X^{(g)})^T W r^{(-g)}\|_2 > \lambda_2\alpha\sqrt{p_g} \\ 0 & \text{if } \|(X^{(g)})^T W r^{(-g)}\|_2 \leq \lambda_2\alpha\sqrt{p_g} \end{cases} \quad (25)
$$

An assumption that is often made in group lasso problems is the within-group orthonormality, where $(X^{(g)})^T X^{(g)} = I$, so that (25) has closed form solution[2,4]. For our case, this orthonormality does not necessarily hold. Therefore we solve for the general case. Let $Q^{(g)} = W^{1/2}X^{(g)} = (diag\{\sqrt{w_1}, ..., \sqrt{w_N}\})X^{(g)}$, and $\mu^{(-g)} = W^{1/2}r^{-g}$, then we rewrite (25) as

$$\tilde{\beta}^{(g)} = \begin{cases} \Big((Q^{(g)})^T Q^{(g)} + \lambda_2\big[(1-\alpha) + \frac{\alpha\sqrt{p_g}}{\|\tilde{\beta}^{(g)}\|}\big] I^{(g)}\Big)^{-1} (Q^{(g)})^T \mu^{(-g)} & \text{if } \|(Q^{(g)})^T \mu^{(-g)}\|_2 > \lambda_2 \alpha \sqrt{p_g} \\ 0 & \text{if } \|(Q^{(g)})^T \mu^{(-g)}\|_2 \le \lambda_2 \alpha \sqrt{p_g} \end{cases} \tag{26}$$

For the case of $\|(Q^{(g)})^T \mu^{(-g)}\|_2 \le \lambda_2 \alpha \sqrt{p_g}$, we have explicit solution that $\tilde{\beta}^{(g)} = 0$. Therefore we focus on the case of $\|(Q^{(g)})^T \mu^{(-g)}\|_2 > \lambda_2 \alpha \sqrt{p_g}$. Notice that if we know the ℓ_2-norm $\|\beta^{(g)}\|$, then (26) becomes closed form solution. Therefore, we first find the norm $\|\beta^{(g)}\|$. We take the singular value decomposition $Q^{(g)} = U^{(g)} D^{(g)} (V^{(g)})^T$, where $U^{(g)}$ and $V^{(g)}$ has orthonormal columns, and $D^{(g)} = diag\{d_1^{(g)}, ..., d_{p_g}^{(g)}\}$ is diagonal matrix. Let $\eta^{(-g)} = [\eta_1^{(-g)}, ..., \eta_{p_g}^{(-g)}]^T = (U^{(g)})^T \mu^{(-g)}$. For simplicity of notations, we are omitting superscript '(g)' in U,D and V, but keep in mind that we are solving for an individual group g. As a result, for (26), we have

$$\tilde{\beta}^{(g)} = \Big((Q^{(g)})^T Q^{(g)} + \lambda_2\big[(1-\alpha) + \frac{\alpha\sqrt{p_g}}{\|\tilde{\beta}^{(g)}\|}\big] I^{(g)}\Big)^{-1} (Q^{(g)})^T \mu^{(-g)} \tag{27}$$

$$\iff \quad \tilde{\beta}^{(g)} = \Big(V D^2 V^T + \lambda_2 V\big[(1-\alpha) + \frac{\alpha\sqrt{p_g}}{\|\tilde{\beta}^{(g)}\|}\big] I^{(g)} V^T\Big)^{-1} V D U^T \mu^{(-g)} \tag{28}$$

$$\iff \quad V^T \tilde{\beta}^{(g)} = \Big(D^2 + \lambda_2\big((1-\alpha) + \frac{\alpha\sqrt{p_g}}{\|\tilde{\beta}^{(g)}\|}\big) I\Big)^{-1} D \eta^{(-g)} \tag{29}$$

Note that LHS and RHS of (29) are two vectors, and take ℓ_2-norm on both sides, we get

$$\|\tilde{\beta}^{(g)}\|_2^2 = \sum_{i=1}^{p_g} \frac{d_i^2 \eta_i^2}{\big(d_i^2 + \lambda_2(1-\alpha) + \lambda_2 \frac{\alpha\sqrt{p_g}}{\|\tilde{\beta}^{(g)}\|}\big)^2} \tag{30}$$

$$\iff \quad \sum_{i=1}^{p_g} \frac{d_i^2 \eta_i^2}{\big([d_i^2 + \lambda_2(1-\alpha)]\|\tilde{\beta}^{(g)}\|_2 + \lambda_2 \alpha \sqrt{p_g}\big)^2} = 1 \tag{31}$$

Therefore, the ℓ_2-norm of the optimal solution, $\|\tilde{\beta}^{(g)}\|_2$, is the solution to equation $f(\gamma) = 0$, which is

$$f(\gamma) = \sum_{i=1}^{p_g} \frac{d_i^2 \eta_i^2}{\big([d_i^2 + \lambda_2(1-\alpha)]\gamma + \lambda_2 \alpha \sqrt{p_g}\big)^2} - 1 = 0 \tag{32}$$

It is easy to check that f is convex, $f(0) > 0$, and f is monotonically decreasing as γ increases, and $\lim_{\gamma \to \infty} f(\gamma) = -1$. Therefore, $\|\tilde{\beta}^{(g)}\|_2$ is the only solution to $f(\gamma) = 0$. And $\|\tilde{\beta}^{(g)}\|_2$ can be efficiently found by Newton's method.

Once we find the optimal $\|\tilde{\beta}^{(g)}\|_2$, (26) becomes the closed form solution, and we can solve (14) using block coordinate descent. The algorithm can be summarized as in Algorithm 2.

Note that Algorithms 1 and 2 are effectively second-order methods with a fixed step size of 1. It is also possible to adapt it into a backtrack line search to find an optimal step length adaptively.

Algorithm 2: Solve the group elastic-net regularized GLM using coordinate descent

Data: data matrix $X_{pre} \in \mathbb{R}^{N \times T_1}$ for pre-stimulus part of the data, and the corresponding group partition into G groups, with group length $\{p_1, ..., p_G\}$, data label $y \in \mathbb{R}^N$ and fixed post-stimulus solution β^*_{post}; where N is the number of samples, $T_1 = t^P_{pre} + t^{BHA}_{pre} + t^{phase}_{pre}$, and $X_{pre} = [X^P_{pre}, X^{BHA}_{pre}, X^{phase}_{pre}]$;

Parameters: the elastic-net hyper-parameter α, maximum regularization parameter λ_{max} and minimum regularization parameter $\epsilon\lambda_{max}$.

Result: Weight vectors for post-stimulus features $\beta^*_{pre} = [\beta^{pre}_0, \beta^P_{pre}, \beta^{BHA}_{pre}, \beta^{phase}_{pre}]$

Fit the group elastic-net problem for pre-stimulus features:

for *the i-th cross-validation split* $\{X^{(i)}_{pre,train}, X^{(i)}_{pre,test}\}$ **do**

    for $\lambda \leftarrow \lambda_{max}$ **to** $\epsilon\lambda_{max}$ *(decrement* λ*)* **do**

        while *not converge* **do**

            update the current quadratic approximation (22) by computing (19),(20);

            for $j \leftarrow 1$ **to** G *(cyclic block coordinate descent)* **do**

                use Newton's method to solve for the norm $\|\tilde{\beta}^{(g)}\|$ in equation (32);

                update the weight of each coordinate group $\tilde{\beta}^{(g)}$ using $\|\tilde{\beta}^{(g)}\|$ and (26) ;

        estimate the deviance of the solution for current λ on $X^{(i)}_{pre,test}$;

find optimal λ^* and the corresponding β^*_{pre} that minimizes deviance.

Supplementary References:

1 Ghuman, A. S. *et al.* Dynamic encoding of face information in the human fusiform gyrus. *Nat Commun* **5**, 5672, doi:10.1038/ncomms6672 (2014).

2 Hastie, T., Tibshirani, R., & Wainwright, M., *Statistical learning with sparsity: the lasso and generalizations*. (CRC press, 2015).

3 Breheny, P. & Huang, J. Group descent algorithms for nonconvex penalized linear and logistic regression models with grouped predictors. *Stat Comput* **25**, 173-187, doi:10.1007/s11222-013-9424-2 (2015).

4 Simon, N., & Tibshirani, R. Standardization and the group lasso penalty. *Statistica Sinica* **22**, 983-1001 (2012).
