# Supplementary material for: Endogenous activity modulates stimulus and circuit-specific neural tuning and predicts perceptual behavior
Source: Nat Commun. 2020 Aug 11;11:4014. doi: 10.1038/s41467-020-17729-w (PMC7419548; doi:10.1038/s41467-020-17729-w)
Supplement: Supplementary file 3 — Reporting Summary [file 41467_2020_17729_MOESM3_ESM.pdf]

nature research

Corresponding author(s): Yuanning Li

Last updated by author(s): Jun 4, 2020

Reporting Summary

Nature Research wishes to improve the reproducibility of the work that we publish. This form provides structure for consistency and transparency in reporting. For further information on Nature Research policies, see Authors & Referees and the Editorial Policy Checklist .

Statistics

For all statistical analyses, confirm that the following items are present in the figure legend, table legend, main text, or Methods section.

n/a	Confirmed	
☐	☒	The exact sample size (*n*) for each experimental group/condition, given as a discrete number and unit of measurement
☐	☒	A statement on whether measurements were taken from distinct samples or whether the same sample was measured repeatedly
☐	☒	The statistical test(s) used AND whether they are one- or two-sided<br>*Only common tests should be described solely by name; describe more complex techniques in the Methods section.*
☐	☒	A description of all covariates tested
☐	☒	A description of any assumptions or corrections, such as tests of normality and adjustment for multiple comparisons
☐	☒	A full description of the statistical parameters including central tendency (e.g. means) or other basic estimates (e.g. regression coefficient) AND variation (e.g. standard deviation) or associated estimates of uncertainty (e.g. confidence intervals)
☐	☒	For null hypothesis testing, the test statistic (e.g. *F*, *t*, *r*) with confidence intervals, effect sizes, degrees of freedom and *P* value noted<br>*Give P values as exact values whenever suitable.*
☒	☐	For Bayesian analysis, information on the choice of priors and Markov chain Monte Carlo settings
☒	☐	For hierarchical and complex designs, identification of the appropriate level for tests and full reporting of outcomes
☐	☒	Estimates of effect sizes (e.g. Cohen's *d*, Pearson's *r*), indicating how they were calculated

Our web collection on statistics for biologists contains articles on many of the points above.

Software and code

Policy information about availability of computer code

Data collection	The experiment paradigm was performed using custom MATLAB (R2009a) code and Psychtoolbox 3.
Data analysis	The data analysis was performed using FreeSurfer 6, BrainStorm 3, and custom code in MATLAB (R2017a) and R (ver3.4.3).

For manuscripts utilizing custom algorithms or software that are central to the research but not yet described in published literature, software must be made available to editors/reviewers. We strongly encourage code deposition in a community repository (e.g. GitHub). See the Nature Research guidelines for submitting code & software for further information.

Data

Policy information about availability of data

All manuscripts must include a data availability statement. This statement should provide the following information, where applicable:

- Accession codes, unique identifiers, or web links for publicly available datasets
- A list of figures that have associated raw data
- A description of any restrictions on data availability

The data and code that support the findings of this study are available from the corresponding author upon reasonable request.

Field-specific reporting

Please select the one below that is the best fit for your research. If you are not sure, read the appropriate sections before making your selection.

☒ Life sciences ☐ Behavioural & social sciences ☐ Ecological, evolutionary & environmental sciences

For a reference copy of the document with all sections, see nature.com/documents/nr-reporting-summary-flat.pdf

Life sciences study design

All studies must disclose on these points even when the disclosure is negative.

Sample size	No explicit sample size calculation was performed. The amount of data collected from each participant was dependent on their clinical treatment schedule and the amount of time each participant was willing to volunteer for the study. The fact that categorical sensitive electrodes can be identified from all participants suggests that the amount of repetitions within each task block is sufficient. The fact that we found positive pre-stimulus modulation effect from all visual categories indicated that the total amount of participants is sufficient.
Data exclusions	No data were excluded from analysis.
Replication	All classification results were evaluated on held-out testing data using 5-fold cross-validation (5 times). All replications were successful.
Randomization	The images were presented in random order in each task block.
Blinding	Blinding was not relevant for this study. The participants' task was to detect repetitions in the series of images being presented, which could not bias any analysis or results that we reported.

Reporting for specific materials, systems and methods

We require information from authors about some types of materials, experimental systems and methods used in many studies. Here, indicate whether each material, system or method listed is relevant to your study. If you are not sure if a list item applies to your research, read the appropriate section before selecting a response.

Materials & experimental systems

n/a	Involved in the study	
☒	☐	Antibodies
☒	☐	Eukaryotic cell lines
☒	☐	Palaeontology
☒	☐	Animals and other organisms
☐	☒	Human research participants
☒	☐	Clinical data

Methods

n/a	Involved in the study	
☒	☐	ChIP-seq
☒	☐	Flow cytometry
☒	☐	MRI-based neuroimaging

Human research participants

Policy information about studies involving human research participants

Population characteristics	30 human subjects (11 male, 19 female) were recruited. All were diagnosed with intractable epilepsy and underwent surgical placement of subdural electrocorticographic electrodes or stereotactic electroencephalography as standard of care for seizure onset zone localization. The ages of the subjects ranged from 19 to 64 years old (mean = 38.2, SD = 11.9). Due to this data collection setup, no covariates or participant properties could be controlled for.
Recruitment	Participants were neurosurgical patients undergoing surgical treatment for intractable epilepsy at UPMC. Participants were invited to participate in the research after they decided to undergo treatment. Their decision to participate in the research was strictly voluntary and had no effect on their clinical treatment. Since the participants' medical condition has had no impact on their ability to perform cognitive tasks, we do not expect there to be any selection bias and we believe them to be representative of general population in terms of visual cognition.
Ethics oversight	The experimental protocols were approved by the Institutional Review Board of the University of Pittsburgh.

Note that full information on the approval of the study protocol must also be provided in the manuscript.
